# Growth and physiological adaptation of whole plants and cultured cells from a halophyte turf grass under salt stress

**DOI:** 10.1093/aobpla/plu041

**Published:** 2014-08-18

**Authors:** Yuichi Tada, Shiho Komatsubara, Takamitsu Kurusu

**Affiliations:** 1 School of Bioscience and Biotechnology, Tokyo University of Technology, 1404-1 Katakura, Hachioji, Tokyo 192-0982, Japan; 2 Graduate School of Bionics, Tokyo University of Technology, 1404-1 Katakura, Hachioji, Tokyo 192-0982, Japan

**Keywords:** Chloride ion, cultured cells, halophyte, potassium ion, proline, salt tolerance, sodium ion, *Sporobolus virginicus*

## Abstract

Understanding the mechanisms used by halophytic members of the Poaceae to cope with salt stress will contribute to the knowledge necessary to genetically engineer salt-tolerant crops. In this study, we identified a genotype of *Sporobolus virginicus*, a halophytic turf grass collected in Japan, and investigated its growth rate, ion concentration and secretion, and proline concentration in comparison with the reported properties of genotypes collected from the USA, South Africa and Egypt. Surprisingly, the Japanese genotype showed a salinity tolerance up to 1.5 M NaCl, a 3-fold higher concentration than seawater salinity. Shoot growth was stimulated by 100 mM NaCl and root growth was stimulated at salinities of up to 1 M NaCl. Accumulation of Na^+^ and CI^−^ in shoots and roots was rapidly elevated by salinity stress but did not exceed the levels required for osmotic adjustment, due in part to ion secretion by salt glands, which are present in genotypes of *S. virginicus*. However, the Japanese genotypes accumulated K^+^ to a higher level than other genotypes, resulting in a relatively high K^+^/Na^+^ ratio even under salinity stress. An increase in proline concentration was observed that was proportional to the NaCl concentration in the culture solution and might partially account for osmotic adjustment in the shoots. We also generated and characterized cultured cells of *S. virginicus*. In 500 mM NaCl, the cultured cells showed an enhanced growth compared with cultured cells of rice. The concentration of Na^+^ and CI^−^ in the cultured cells in 300–500 mM NaCl was lower than in 100 mM NaCl. Cultured cells of *S. virginicus* accumulated proline to higher levels than rice cells cultured under salinity stress. The active regulation of Na^+^, Cl^−^ and K^+^ influx/efflux and proline accumulation might be involved in salt tolerance mechanisms at the cellular level as well as *in planta*.

## Abstract (precis)

Soil salinization is a serious problem in agricultural lands worldwide. Understanding the mechanisms of salt-tolerant plants will contribute to knowledge necessary to genetically engineer salt-tolerant crops that grow on these saline lands. We identified a genotype of *Sporobolus virginicus*, a salt-tolerant turf grass, that showed a salinity tolerance to up to a three-fold higher NaCl concentration than seawater salinity. In addition to salt secretion from salt glands on the leaves, this genotype accumulated K^+^ and proline, a compatible solute, to higher levels than other genotypes under salinity. These properties must contribute to the advanced salt tolerance of this genotype.

## Introduction

Halophytes are plants that can survive high concentrations of electrolytes in their environments and are unique in their ability to accumulate concentrations of salts in their leaves that equal or exceed those of seawater without detrimental effects (Flowers *et al.* 1977). In these plants, the accumulation of ions and compatible solutes is crucial to balance the cytoplasmic osmotic potential with the low water potentials in their environments and to maintain shoot succulence (Flowers *et al.* 1977). In some species, excess ions are secreted through salt glands on the leaf surface (Liphschtz and Waisel 1974). Thus, the mechanisms that mediate salt tolerance in halophytes are partially known; however, specific responses to salinity stress in each halophyte still remain to be elucidated. Understanding the mechanisms to cope with salt stress will contribute to the knowledge necessary to genetically engineer salt-tolerant plants.

*Sporobolus virginicus* is a halophytic C_4_ grass found worldwide, from tropical to warm temperate regions (Blits and Gallagher 1991) that secretes excess salt from salt glands on the leaf surface (G. Naidoo and Y. Naidoo 1998; Y. Naidoo and G. Naidoo 1998). Growth under saline conditions has been studied in ecotypes/genotypes of *S. virginicus* collected from populations in Georgia, Florida and Hawaii, USA (Gallagher 1979; Blits and Gallagher 1991; Marcum and Murdoch 1992; Bell and O'Leary 2003), Durban, South Africa (Breen *et al.* 1977; Naidoo and Mundree 1993; G. Naidoo and Y. Naidoo 1998; Y. Naidoo and G. Naidoo 1998) and Egypt (Ashour *et al.* 1997). Growth and ion accumulation differ among these genotypes of *S. virginicus* collected from different locations, although the source of plant material and growth conditions differed in each study.

In this study, we collected a genotype of *S. virginicus* inhabiting the sea shore at Iriomote Island, Okinawa, Japan, and examined the growth and physiological properties, including average daily growth rate, ion concentration, ion secretion and proline accumulation under saline conditions. Proline accumulation is one of the most frequently reported modifications induced by salinization and drought in plants, and is often considered to be involved in stress-tolerance (Delauney and Verma 1993; Hare *et al.* 1999). We also established cultured cells of *S. virginicus* to investigate whether the salt-induced responses occur at the cellular level and compared the cellular response to salt stress with that of a glycophyte, rice. A comparison of undifferentiated cell cultures to differentiated whole plants can help to determine the level of cellular organization necessary to invoke a particular response to salt (Thomas *et al.* 1992). A comparison of halophytic cells with glycophytic cells can help to determine the cellular mechanisms in halophytes that are necessary to cope with extracellular salt stress.

## Methods

### Plant growth and salt treatments

Rhizomes of *S. virginicus* were collected from Iriomote Island, Okinawa, Japan, and grown in culture pots filled with ‘Akadama’ soil (red clay ball) or vermiculite in a purpose-built growth room (SANYO Electric Co. Ltd, Tokyo, Japan) at 27 °C, 70 % humidity and a 12 h light/12 h dark cycle with a photon-flux density of 350 μmol photons m^−2^ s^−1^ under white florescent lights. Stress treatment of potted plants was carried out by immersing the pots in 0, 500, 1000, 1500 or 2000 mM NaCl solution. For hydroponic culture, stolons of *S. virginicus* were cut, rooted and cultivated in the hydroponic system ‘Home Hyponica 501’ or ‘Home Hyponica 601’ using Hyponica culture solution (Kyowa Co. Ltd, Osaka, Japan), which contains 80 mg L^−1^ N, 76 mg L^−1^ P, 188 mg L^−1^ K and minor elements. Plants of a similar size and weight (880 mg on average) were selected for stress treatment. All plants were washed as below with deionized water to remove the previously secreted salts at the beginning of the stress treatment and were then wiped with a paper towel. Stress treatment of plants in hydroponic culture was carried out by transferring the plants to Hyponica culture solution supplemented with 0, 500, 750, 1000, 1250 or 1500 mM NaCl. To minimize osmotic shock, for salinity treatments >500 mM NaCl, salinity was increased from 500 mM at the start, by 250 mM increments at 3–4-day intervals until the final level was reached. Solutions were changed every 2 weeks during experiments. To determine the average daily growth rate, shoots and roots were sampled 6 weeks after stress treatment. Plants were washed briefly in 2 L of deionized water in a beaker, wiped with a paper towel and the dry weight (DW) then determined after drying fresh samples for 24–48 h in an oven at 60 °C. The average daily growth rate (mg day^−1^) was calculated as (DW after the treatment−DW before the treatment)/42. To determine ion and proline concentrations, the shoots and roots were sampled from hydroponically cultured plants at 0, 1, 3, 7, 14 and 21 days after NaCl treatment and their DW determined before ion and proline determination. The roots were washed briefly in water and wiped with paper, to eliminate the transfer of salt from culture solution. To determine the rate of sodium secretion, the shoots sampled at each time point were inserted into 50-mL sample tubes with 10 mL of deionized water, and immediately shaken for 1 min to dissolve salt secreted from the salt glands. The extract was then subjected to ion analysis and shoots were paper-wiped, weighed and allowed to dry at 60 °C for 24 h to determine DW. Care was taken not to disturb the leaf surface during harvesting of the leaves.

### Cell cultures

To generate axenic plants of *S. virginicus*, stolons were cut from potted plants and surface-sterilized with 1 % NaClO for 30 min, rinsed three times with sterile water and cultured in a plant culture box ‘Magenta GA-7’ (PlantMedia, Dublin, OH, USA) with medium containing 1/2 Murashige and Skoog (MS) salts (Murashige and Skoog 1962), 1 % sucrose and 0.2 % gellan gum, pH 5.5. Roots were cut from these sterile plants and placed on medium containing 1/2 MS salts, Gamborg's B-5 vitamins (Gamborg *et al.* 1968), 1 % sucrose, 1.0 ppm 2,4-d, 0.1 ppm BAP and 0.2 % gellan gum, pH 5.5. After 1 month of cultivation, a suspension culture was initiated by transferring calli formed from roots into 20 mL of liquid culture medium consisting of 1/2 MS salts, Gamborg's B-5 vitamins, 1 % sucrose, 1.0 ppm 2,4-d and 0.1 ppm BAP, pH 5.5 in 100 mL Erlenmeyer flasks. Flasks were maintained on a shaker at 100 rpm. Once established, suspension cultures were sub-cultured weekly. The culture condition was 25 °C under 12 h/12 h light/dark cycles.

Induction of callus from matured seeds of rice cultivar ‘Nipponbare’ and establishment of the cultured cells was performed according to Akagi *et al.* (1989). For salt treatments, cultured cells were transplanted into liquid culture medium supplemented with 0, 100, 300 or 500 mM NaCl. Cultured cells were collected by filtering through a nylon mesh (100-μm mesh size). While cells were wrapped in the nylon mesh, they were briefly rinsed in 200 mL of deionized water in a plastic container, and dried with paper towels for 2 min. A portion of cells was then subjected to proline measurement, and the remaining cells were dried at 60 °C for 24 h to determine the DW. A portion of the dried material was used to determine ion concentrations.

### Measurement of ion concentration

To measure ion concentration, the dried plant materials were powdered using a mortar and pestle or a hand crusher ‘SK mill’ (Tokken, Noda, Japan), then were suspended in 0.5 M HNO_3_ or deionized water (20 mg mL^−1^) for cation or anion extraction, respectively, and the mixture was incubated at 80 °C overnight. The extract from dried plants and the leaf surface were diluted with deionized water and filtered using a cellulose acetate filter (0.45-μm pore size). The concentrations of Na^+^, K^+^ and Cl^−^ ions in the extract were determined using an Ion analyzer IA-300 (TOA DKK, Tokyo, Japan). The measurement was performed according to Miyama and Tada (2011). The ion concentrations are expressed as micromoles per gram DW (μmol g DW^−1^).

### Measurement of proline concentration

Fresh or frozen plant material was homogenized using a ‘Magic Bullet’ homogenizer (Homeland Housewares, Los Angeles, CA, USA) for shoots and roots and a ‘SK mill’ for cultured cells and proline concentration was determined by using the method of Bates *et al.* (1973).

### Statistical analyses

Data were analysed by one-way analysis of variance (ANOVA) for comparison of means at different NaCl concentrations. If there were significant differences among the groups, a specific significance test was performed using Tukey's multiple comparison test at the 0.05 probability level. For comparison of differences between means at specific dates and initial (0 day) measurements, data were analysed using a Student's *t*-test at the 0.05 or 0.01 probability level following an *F*-test of equality of variances.

## Results

### Growth of *S. virginicus* plants under salt stress

**Figure 1.**
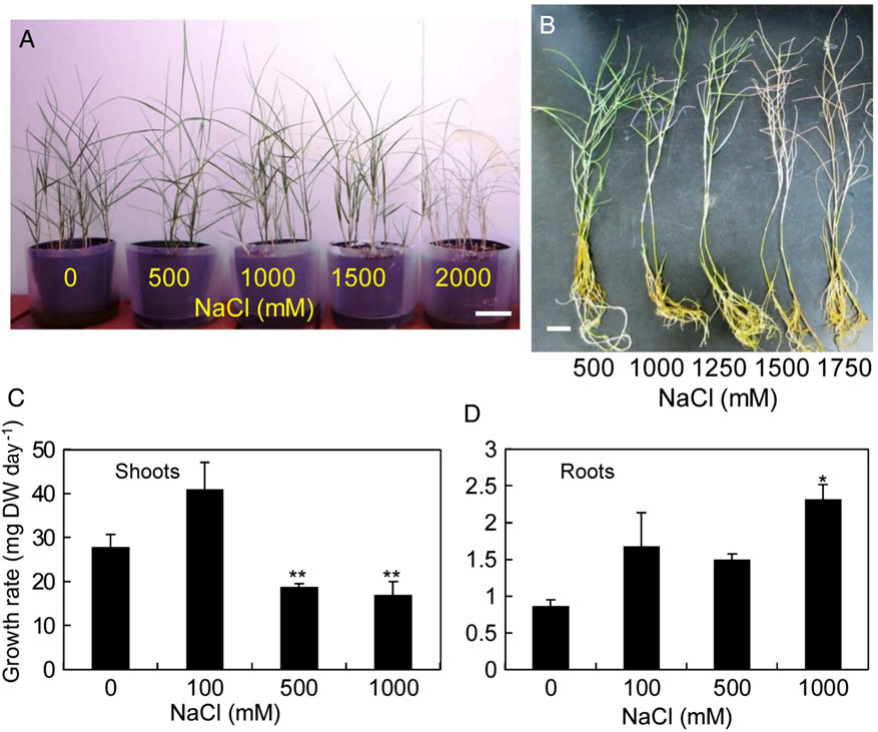
Growth of *S. virginicus* plants at different NaCl concentrations. *S. virginicus* plants cultivated in soil or hydroponic culture were treated with different NaCl concentrations and the average daily growth rate of shoots and roots was determined. (A) Potted plants were treated with 0, 500, 1000, 1500 or 1500 mM NaCl. (B) Hydroponically cultured plants were treated with 0, 500, 1000, 1250, 1500 or 1750 mM NaCl. The average daily growth rate of shoots (C) and roots (D) was determined after 6 weeks of cultivation in solution with 0, 100, 500 or 1000 mM NaCl. Scale = 5 cm. Single and double asterisks denote significant differences from values at 0 mM NaCl at *P* < 0.05 and 0.01, respectively, using Student's *t*-test.

The salt tolerance of the Japanese genotype of *S. virginicus* was examined in both soil and hydroponic culture solution supplemented with different concentrations of NaCl. When cultivated in soil, *S. virginicus* plants could survive up to 1.5 M NaCl, but died in 2 M NaCl after 6 weeks following treatment (Fig. 1A). No visible damage was observed to the plants in 1.5 M NaCl. Similar experiments using Akadama (three times) and vermiculite (twice) were performed with similar results (data not shown). When hydroponically cultured, *S. virginicus* plants survived up to 1.25 M NaCl (Fig. 1B). In hydroponic culture solution supplemented with 0, 100, 500, or 1000 mM NaCl, the average daily growth rates of shoots and roots was determined after 6 weeks of cultivation (Fig. 1C and D). The shoot growth rate was promoted in 100 mM NaCl (40 mg DW day^−1^) compared with the controls (27 mg DW day^−1^), but was significantly lower in the 500 and 1000 mM NaCl treatments (18 and 15 mg DW day^−1^). In contrast, roots showed stimulated growth at all salt levels. In particular, root growth was significantly enhanced in 1000 mM NaCl (2.3 mg DW day^−1^) compared with that in the control treatment (0.8 mg DW day^−1^). No visible damage was observed up to 500 mM NaCl, although slight wilting was seen with 1000 mM NaCl.

### Ion concentrations in *S. virginicus* plants

**Figure 2.**
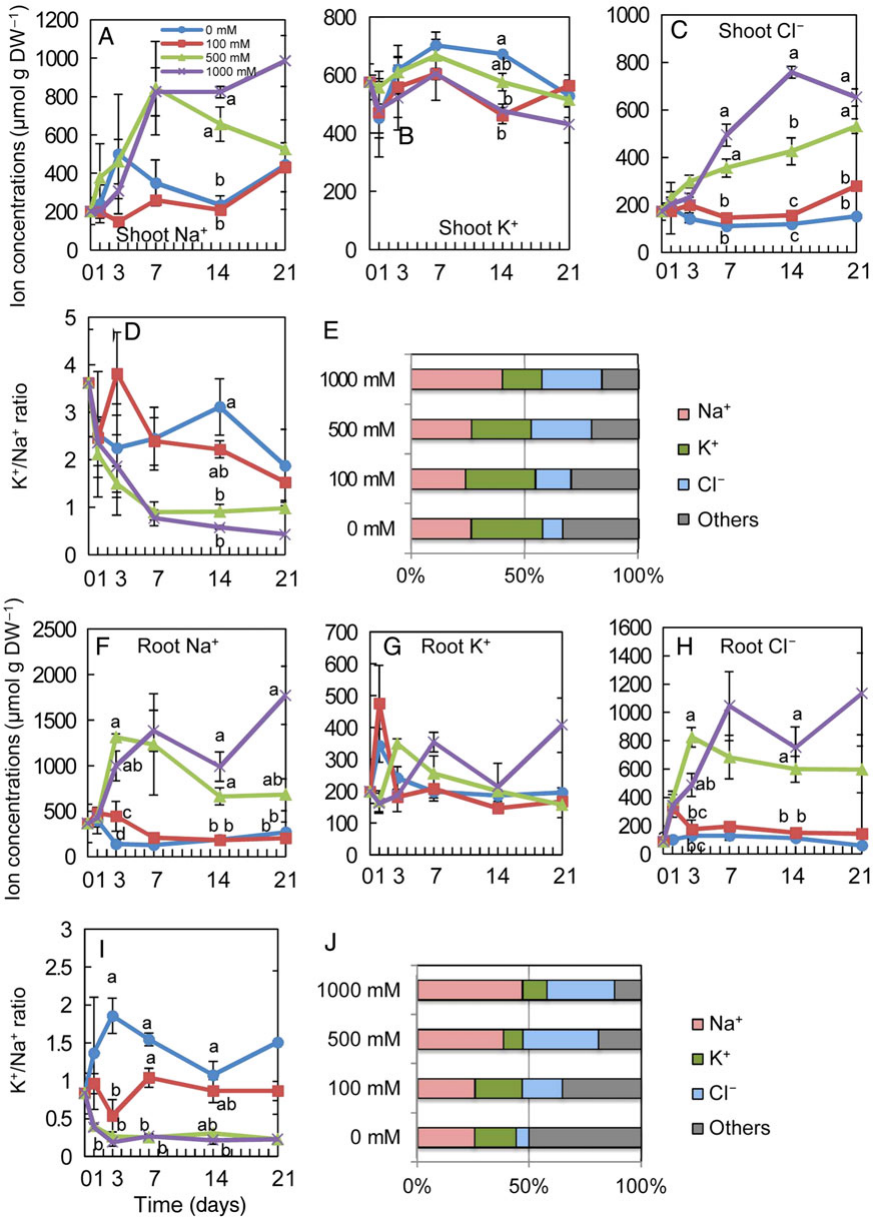
Ion concentration of *S. virginicus* plants under different NaCl concentrations. Changes in shoot Na^+^ (A), K^+^ (B) and Cl^−^ (C) concentration, the K^+^/Na^+^ ratio (D), root Na^+^ (F), K^+^ (G) and Cl^−^ (H) concentrations and the K^+^/Na^+^ ratio (I) at 0, 1, 3, 7, 14 and 21 days after salt treatments, and the molar ratio of each ion of the total ion concentration in shoots (E) and roots (J) at 21 days after salt treatment. Means with a different letter for the same day are significantly different at *P* < 0.05 using Tukey's method.

To investigate ion accumulation into and ion exclusion from *S. virginicus*, plants were cultivated hydroponically in solution supplemented with 0, 100, 500 or 1000 mM NaCl for 3 weeks and ion concentrations in shoots and roots were determined (Fig. 2). In both shoots and roots, accumulation of Na^+^ and CI^−^ was rapidly elevated by salinity, but stayed almost constant or decreased after reaching the maximum levels (Fig. 2A, C, F and H). Shoots maintained a higher K^+^ concentration than roots under all NaCl levels (Fig. 2B and G). As a consequence, the K^+^/Na^+^ ratio in the shoots remained at 1.6–3.8 under moderate (0 and 100 mM) NaCl conditions, and at 0.5–1.0 in the 500 and 1000 mM NaCl treatments (Fig. 2D). In the roots, the K^+^/Na^+^ ratio was lower than that of shoots at all NaCl levels. An increase in Cl^−^ concentration in shoots with increasing NaCl concentration in culture solution and culture period was observed at 500 and 1000 mM NaCl (Fig. 2C). Roots accumulated Cl^−^ at a higher level than shoots at 500 and 1000 mM NaCl. The quantity of Na^+^ and K^+^ ions contributed more than 50 % of the measured ions in shoots in the all NaCl treatments tested but less in roots (Fig. 2E and J).

### Secretion of salt from salt glands of *S. virginicus* plants

**Figure 3.**
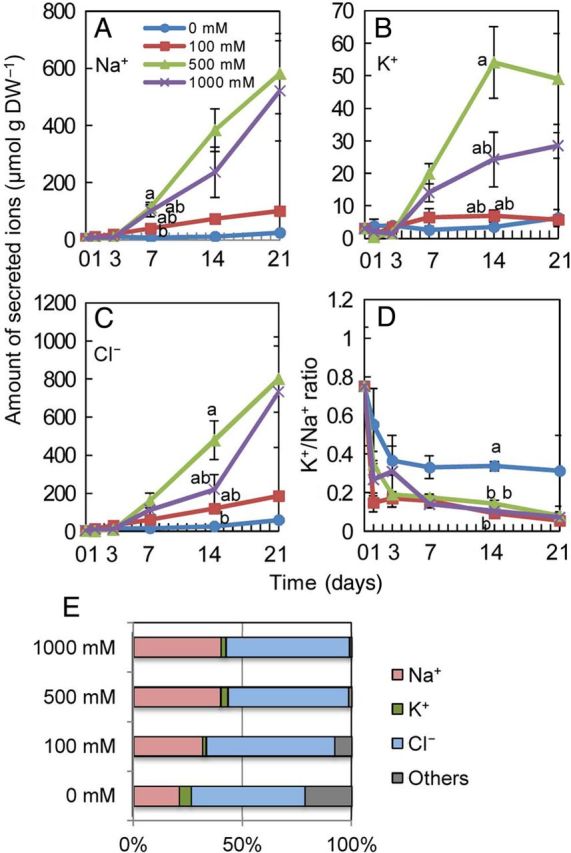
Amount of secreted ions from salt glands on the leaves of *S. virginicus* under different NaCl concentrations. Salts on the leaves of *S. virginicus* were dissolved in deionized water and the amount of secreted Na^+^, K^+^ and Cl^−^ were determined. Changes in secreted Na^+^ (A), K^+^ (B) and Cl^−^ (C) ions and the K^+^/Na^+^ ratio (D) at 0, 1, 3, 7, 14 and 21 days after salt treatments, and the ratio of each ion in the amount of total secreted ions (E) at 21 days after salt treatment. Means with different letters for the same day are significantly different at *P* < 0.05 using Tukey's method.

We monitored salt secretion from the salt glands of leaves from plants hydroponically cultivated in solutions differing in salinity (Fig. 3). The amount of Na^+^, Cl^−^ and K^+^ that was secreted increased together with an increase in the concentration of NaCl applied, except for the 1000 mM NaCl treatment (Fig. 3A–C). The amount of secreted ions with 1000 mM NaCl was less than that with 500 mM. The total secretion of Na^+^ and Cl^−^ increased according to the culture period (Fig. 3A and C); however, the secretion of K^+^ did not increase after 14 days (Fig. 3B). The K^+^/Na^+^ ratio of secreted ions was similar among all salinity conditions (Fig. 3D). The molar ratio of the Cl^−^ to the total ionic amount was almost constant at 52–59 % at all salinity levels, but that of Na^+^ almost doubled from 21 % at 0 mM NaCl to 40 % at 500 and 1000 mM NaCl (Fig. 3E).

### Growth and ion concentration of cultured cells under salt stress

**Figure 4.**
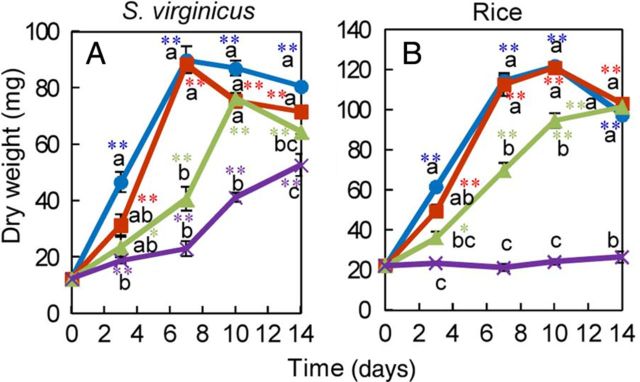
Growth of cultured cells from *S. virginicus* and rice under different NaCl concentrations. Cells from *S. virginicus* and rice were cultured in liquid medium supplemented with different NaCl concentrations and their DWs were determined at 0, 3, 7, 10 and 14 days after subculture. The DW of *S. virginicus* cultured cells (A) and rice cultured cells (B). Means with different letters for the same day are significantly different at *P* < 0.05 using Tukey's method. Single and double asterisks denote significant differences from values at 0 days at *P* < 0.05 and 0.01, respectively, using Student's *t*-test.

To compare the response of cultured cells with whole plants of *S. virginicus* and the response of *S. virginicus* with rice at a cellular level in different salinity treatments, cells of both species were cultured in liquid medium with different NaCl concentrations and their DWs were measured for 2 weeks. The DW of *S. virginicus* and rice cultured cells was similar at 0, 100 and 300 mM NaCl (Fig. 4A and B); however, *S. virginicus* cultured cells, but not those of rice, increased in DW even in the 500 mM NaCl treatment.

**Figure 5.**
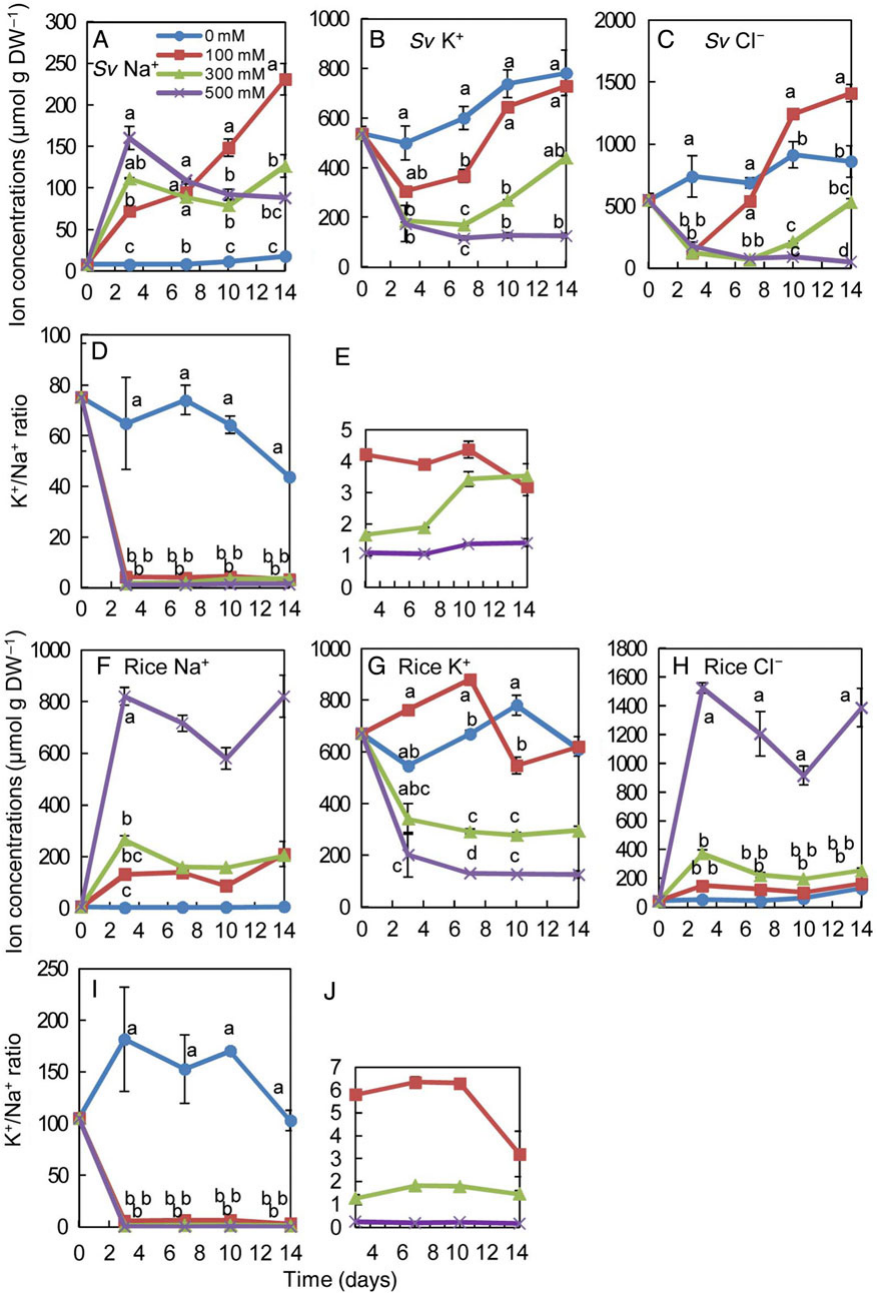
Ion concentration and ratios in cultured cells of *S. virginicus* and rice under different NaCl concentrations. Changes in Na^+^ (A), K^+^ (B) and Cl^−^ (C) concentration and the K^+^/Na^+^ ratio in *S. virginicus* cultured cells (D and E) and Na^+^ (F), K^+^ (G) and Cl^−^ (H) concentration and the K^+^/Na^+^ ratio (I and J) in rice cultured cells at 0, 3, 7, 10 and 14 days after salt treatments. Panels (E) and (J) are enlarged graphs of values for 100–500 mM NaCl in (D) and (I), respectively. Means with different letters for the same day are significantly different at *P* < 0.05 using Tukey's method.

The ion concentrations in cultured cells of *S. virginicus* were very different from those in shoots and roots (Fig. 5). Cultured cells of *S. virginicus* with 300 and 500 mM NaCl accumulated Na^+^ at higher levels, but Cl^−^ at lower levels, than in the 0 mM NaCl treatment during the culture period (Fig. 5A and C). The concentration of K^+^ in *S. virginicus* cultured cells decreased as the NaCl concentration in the medium increased (Fig. 5B). Consequently, a high K^+^/Na^+^ ratio (44–75) in *S. virginicus* cultured cells under non-stress conditions dramatically decreased to a level (1–4) similar to that in the shoots under salinity treatment. In rice cultured cells, the concentration of Na^+^ and Cl^−^ increased as the NaCl concentration in the medium increased, and extreme increases were particularly observed following 500 mM NaCl treatment (Fig. 5F and H). These responses differed greatly from those in *S. virginicus* cultured cells, in which Na^+^ and Cl^−^ concentrations were maintained, even in the 500 mM NaCl treatment. Alterations in the K^+^ concentration of rice cells were similar to those in *S. virginicus* cells (Fig. 5B and G). As a concequence, the K^+^/Na^+^ ratio in rice cultured cells was maintained at a higher level in 0 and 100 mM NaCl, but at a lower level in 300 and 500 mM NaCl than that in *S. virginicus* cultured cells (Fig. 5D, E, I and J), indicating that K^+^/Na^+^ homeostasis in *S. virginicus* cultured cells is more stable than that in rice cultured cells under salinity treatment.

### Proline concentration in whole plants and cultured cells

**Figure 6.**
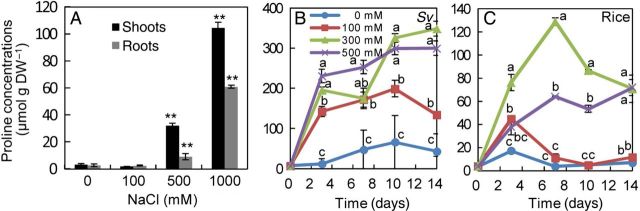
Proline concentrations in shoots, roots and cultured cells of *S. virginicus* and rice cultured cells in different NaCl concentrations. The proline concentration was determined in shoots and roots of *S. virginicus* hydroponically cultivated with 0, 100, 500, 1000 mM NaCl for 21 days (A) and in cultured cells of *S. virginicus* (B) and rice (C) with 0, 100, 300, 500 mM NaCl. Asterisks denote significant differences from values at 0 mM NaCl at *P* < 0.01 using the Student's *t*-test. Means with different letters for the same day are significantly different at *P* < 0.05 using Tukey's method.

The free proline concentration in shoots, roots and cultured cells was determined to investigate the role of proline accumulation in whole plants and cultured cells of *S. virginicus* under salinity treatment. The proline concentration in shoots and roots of *S. virginicus* did not increase with 100 mM NaCl compared with the 0 mM NaCl treatment, but increased by 10- and 40-fold in shoots and by 4- and 34-fold in roots in the 500 and 1000 mM NaCl treatments, respectively (Fig. 6A); thus, shoots accumulate more proline than roots under these conditions.

In cultured cells of *S. virginicus*, the proline concentration increased almost in proportion to an increase in NaCl concentration in the medium (Fig. 6B). At 100 mM NaCl, a concentration that did not induce proline accumulation in shoots and roots, the proline concentration in cultured cells increased to 199 μmol g DW^−1^ at 10 days. The maximum proline concentration of *S. virginicus* cultured cells was 349 μmol g DW^−1^, which was 3.3- and 5.7-fold higher than those in shoots and roots, respectively. In contrast, rice cultured cells accumulated proline at lower levels than *S. virginicus* cultured cells in all treatments (Fig. 6C).

## Discussion

### Salt tolerance of Japanese genotype of *S. virginicus*

The growth of *S. virginicus* has been reported for genotypes/ecotypes collected from populations in Georgia, Florida and Hawaii, USA (Gallagher 1979; Blits and Gallagher 1991; Marcum and Murdoch 1992; Bell and O'Leary 2003), Durban, South Africa (Naidoo and Mundree 1993; G. Naidoo and Y. Naidoo 1998; Y. Naidoo and G. Naidoo 1998) and Egypt (Ashour *et al.* 1997) and grown hydroponically or in soil with different concentrations of NaCl. In our study, we evaluated the growth and physiological adaptation of *S. virginicus* collected at Iriomote Island, Japan, and compared them with the reported properties of the other genotypes (Table 1). Surprisingly, the Japanese genotype survived even in 1.5 M NaCl, a concentration 3-fold higher than seawater salinity. Although the Georgia genotype could tolerate irrigation with water containing up to 8 % (1.38 M) NaCl, the biomass was 3.9 % of that not stressed by NaCl (Gallagher 1979). The maximum salinity concentrations in which the other genotypes survived were not determined, but the Japanese genotype was not at all inferior to the other genotypes in the level of salt it could withstand.

**Table 1.** Growth and physiological properties of *S. virginicus* genotypes collected from different places. ND, not determined.

Origin	Tolerable NaCl concentration (mM)	Growth stimulation with NaCl treatment	Na^+^, Cl^−^ and K^+^ accumulation under high saline condition	K^+^/Na^+^ ratio under seawater	Proline accumulation in shoots (μmol g DW^−1^)
Okinawa, Japan (This study)	1500	Whole plant: 100 mM	Yes	1.0	32.2 and 101.5 at 500 and 1000 mM NaCl, respectively
		Roots: up to 1000 mM			
Georgia, USA (Gallagher 1979)	1380 (8 %)	Not observed	Yes	0.85	ND
Florida, USA (Blits and Gallagher 1991)	ND	Roots: seawater	Yes, but Cl^−^ concentration was ND	1.09–1.39	ND
Hawaii, USA (Marcum and Murdoch 1992)	ND	Shoots: 150 mM	Yes	0.4	22.9 at 450 mM NaCl
		Roots: up to 450 mM			
Hawaii, USA (Bell and O'Leary 2003)	ND	Above- and below-ground biomass: 100–150 mM	Yes, but Cl^−^ concentration was ND	0.3	ND
Durban, South Africa (Naidoo and Mundree 1993; G. Naidoo and Y. Naidoo 1998; Y. Naidoo and G. Naidoo 1998)	ND	Not observed	Yes	ND (2.0 under 80 % seawater)	6.49 at 400 mM NaCl
Egypt (Ashour *et al.* 1997)	ND	Aboveground biomass: up to 125 mM	ND	ND	ND

The growth of the shoot and whole plant of the Japanese genotype was stimulated by 100 mM NaCl, and root growth was stimulated significantly as the concentration of NaCl in the culture solution increased up to 1 M (Fig. 1C and D, Table 1). A larger root mass might be produced in response to high salinity to counter a low external water potential by increasing plant absorptive area (Donovan and Gallagher 1985). Similar growth stimulation under moderate salinity was reported in shoots/whole plant and roots of two Hawaiian genotypes (Bell and O'Leary 2003), in roots of the Florida genotype (Blits and Gallagher 1991) and in shoots of the Egyptian genotype (Ashour *et al.* 1997; Table 1). In contrast, salt-stimulated growth was not observed in studies of genotypes from Georgia (Gallagher 1979) and South Africa (Naidoo and Mundree 1993; G. Naidoo and Y. Naidoo 1998; Y. Naidoo and G. Naidoo 1998). Thus, stimulated growth under moderate salinity does not appear to be a common property among the *S. virginicus* genotypes.

### Regulation of ion concentrations in whole plants

The Japanese genotype accumulated Na^+^ and Cl^−^ when cultivated at 500 and 1000 mM NaCl at levels several folds higher than those grown in the absence of NaCl in both shoots and roots (Fig. 2). These ions accounted for a significant portion of the measured ions in shoots and roots. In the Japanese genotype, the shoot K^+^ concentration was relatively constant, and remained at a relatively high level even in the saline treatments, (Fig. 2B). These K^+^ concentrations were almost 1.5-fold higher than those in the Florida, two Hawaiian and Egyptian genotypes under seawater salinity levels; however, the accumulation of K^+^ in high salinity appeared to be a common property of the *S. virginicus* genotypes (Blits and Gallagher 1991; Marcum and Murdoch 1992; G. Naidoo and Y. Naidoo 1998; Y. Naidoo and G. Naidoo 1998; Bell and O'Leary 2003; Table 1). There were, however, differences in the K^+^/Na^+^ ratio among the genotypes. It was reported that the K^+^/Na^+^ ratios in the leaves or shoots of two Hawaiian and Georgia genotypes grown in seawater salinity levels were 0.85, 0.4 and 0.3, respectively (Gallagher 1979; Marcum and Murdoch 1992; Bell and O'Leary 2003). The Japanese genotype maintained a higher shoot K^+^/Na^+^ ratio (1.0) than those genotypes in seawater salinity, but lower than the ratio (1.09–1.39) in leaves of the Florida genotype in seawater (Blits and Gallagher 1991; Table 1). Low Na^+^ and higher K^+^ levels are typically associated with halotolerant grasses (Breen *et al.* 1977; Gallagher 1979; Flowers and Colmer 2008).

### Secretion of salt from salt glands

The major ions secreted from the salt glands of the Japanese genotype were Na^+^ and Cl^−^ , which is in agreement with data reported for the other genotypes (G. Naidoo and Y. Naidoo 1998; Y. Naidoo and G. Naidoo 1998; Bell and O'Leary 2003). The ratio of these two ions among the total secreted ions increased together with the increased salinity of the culture solution (Fig. 3E) and was higher than the ratio in the shoots (Fig. 2F), indicating the selective secretion of Na^+^ and Cl^−^ over other ions. The molar ratio of Na^+^ among the secreted salts increased together with an increase in salinity in the solution up to 500 mM NaCl (Fig. 3F), whereas the shoot Na^+^ ratio remained almost constant (Fig. 2E). These observations taken together suggest that Na^+^ is dominantly and selectively secreted from leaves. The secretion of Na^+^ might allow *S. virginicus* to maintain transpiration, while avoiding a toxic accumulation of Na^+^.

### Growth and ion concentration of cultured cells under salt stress

The increase in FW of cultured cells from both species was inhibited as the NaCl concentration in the medium increased and little growth was observed in 500 mM NaCl (Fig. 4A). Dry weight of *S. virginicus* cultured cells, however, increased even under 500 mM NaCl (Fig. 4C), whereas that of rice cultured cells did not. Cultured cells of rice varieties have been reported to be much more resistant to salt concentrations that, if applied to whole plants, would kill seedlings (Flowers *et al.* 1985). It is also possible that difference in the explants from which calli were induced also affected salt tolerance of cultured cells from *S. virginicus* and rice. Nevertheless, cultured cells of *S. virginicus* appeared more salt tolerant than those of rice under the saline condition used; the cultured cells, but not those of rice, increased in DW in medium containing 500 mM NaCl.

Because cultured cells of *S. virginicus* were more susceptible to salt stress than the whole plant, salt tolerance (cf. Fig. 1C and D with Fig. 4C) of these halophytes must depend upon the structural and physiological integrity at whole-plant level rather than on cellular mechanisms. However, the fact that the changes in Na^+^ and Cl^−^ concentrations in cultured cells of *S. virginicus* were very different from those in the shoots and roots (Figs. 2A, C, F, H, 5A and C) suggested the existence of distinct ion influx/efflux regulation mechanisms which work at the cellular level, in addition to salt secretion from salt glands on the leaves. Otherwise, different efficiencies or regulation of the genes or proteins involved in the ion influx/efflux may be responsible for the ion balance.

The K^+^/Na^+^ ratio in *S. virginicus* cultured cells was higher than that in rice cells in 300 and 500 mM NaCl treatments (Fig. 5D, E, I and J). The Na^+^ and Cl^−^ concentrations in *S. virginicus* cultured cells were maintained at specific levels in the 300 and 500 mM NaCl treatments, while those in rice cultured cells increased together with an increasing NaCl concentration in the medium, especially under 500 mM NaCl (Fig. 5F and H). Thus, ion homoeostasis was maintained better in *S. virginicus* cultured cells under high salinity than in rice cultured cells, suggesting more active ion regulation in *S. virginicus* cells. Therefore, an analysis of the molecular mechanisms controlling cellular ion influx/efflux in *S. virginicus* would be necessary to reveal the salt tolerance mechanisms.

### Proline concentrations in whole plants and cultured cells

Increased levels of compatible solutes such as proline, glycinebetaine and other polyols following NaCl stress are a common response in both cultured cells and intact plants. Proline accumulation is one of the most frequently reported modifications induced by salt and drought stress in plants, and is often considered to be involved in stress-tolerance mechanisms (Delauney and Verma 1993; Hare *et al.* 1999), recovery from stress and stress signalling (Szabados and Savouré 2010). Proline might interact with enzymes to protect protein structure and activity against the consequences of dehydration-induced thermodynamic perturbation (Naliwajski and Skłodowska 2014). The proline concentrations in shoots of the Japanese genotype were higher than those in South African and Hawaiian genotypes in seawater salinity levels (Fig. 6A; Table 1; Marcum and Murdoch 1992; Naidoo and Mundree 1993; G. Naidoo and Y. Naidoo 1998; Y. Naidoo and G. Naidoo 1998). The greater accumulation of proline might partially contribute to cellular osmotic adjustment of the Japanese genotype in high salinity conditions. Cultured cells of *S. virginicus* accumulated proline to higher levels than rice cultured cells. The proline concentration of cultured cells of the halophyte *Mesembryanthemium crystallinum* and the glycophyte carrot were reported to increase 11.1- and 3.36-fold, respectively, following treatment with 400 mM NaCl (Thomas *et al.* 1992). The fold change of proline (10-fold) in *S. virginicus* shoots following salt treatment was comparable to that in *M. crystallinum*.

## Conclusions

We investigated the growth and physiological adaptation of a genotype of *S. virginicus* collected in Japan in comparison with the reported properties of genotypes collected from the USA, South Africa and Egypt. The Japanese genotype showed a salinity tolerance up to 1.25–1.5 mM NaCl, showing superior salt tolerance of the genotype. The root growth was stimulated at salinities of up to 1 M NaCl. The Japanese genotype accumulated K^+^ to a higher level than other lines, resulting in a relatively high K^+^/Na^+^ ratio even under salinity stress. We also generated and characterized cultured cells of *S. virginicus*. The cultured cells showed an enhanced growth compared with rice cultured cells and accumulated less Na^+^ and Cl^−^ ions and more proline than rice cultured cells under saline conditions. The active regulation of Na^+^, Cl^−^ and K^+^ influx/efflux and proline accumulation might be involved in salt tolerance mechanisms at the cellular level as well as *in planta*.

## Sources of Funding

This work was supported by a grant in aid of the Salt Science Research Foundation to Y.T. (#1312).

## Contributions by the Authors

Y.T. and S.K. designed and conducted the experiment and analysed the data. Y.T. wrote the manuscript. T.K. provided significant experimental and editorial comments.

## Conflicts of Interest Statement

None declared.
